# EasyFlow: An open-source, user-friendly cytometry analyzer with graphic user interface (GUI)

**DOI:** 10.1371/journal.pone.0308873

**Published:** 2024-11-13

**Authors:** Yitong Ma, Inbal Eizenberg-Magar, Yaron Antebi

**Affiliations:** 1 Department of Bioengineering, Stanford University, Stanford, California, United States of America; 2 Department of Molecular Genetics, Weizmann Institute of Science, Rehovot, Israel; Indiana University Bloomington, UNITED STATES OF AMERICA

## Abstract

Flow cytometry enables quantitative measurements of fluorescence in single cells. The technique was widely used for immunology to identify populations with different surface protein markers. More recently, the usage of flow cytometry has been extended to additional readouts, including intracellular proteins and fluorescent protein transgenes, and is widely utilized to study developmental biology, systems biology, microbiology, and many other fields. A common file format (FCS format, defined by the International Society for Advancement of Cytometry (ISAC)) has been universally adopted, facilitating data exchange between different machines. A diverse spectrum of software packages has been developed for the analysis of flow cytometry data. However, those are either 1) costly proprietary softwares, 2) open source packages with prerequisite installation of R or Python and sometimes require users to have experience in coding, or 3) online tools that are limiting for analysis of large data sets. Here, we present EasyFlow, an open-source flow cytometry analysis graphic user interface (GUI) based on Matlab or Python, that can be installed and run locally across platforms (Windows, MacOS, and Linux) without requiring previous coding knowledge. The Python version (EasyFlowQ) is also developed on a popular plotting framework (Matplotlib) and modern user interface toolkit (Qt), allowing more advanced users to customize and keep contributing to the software, as well as its tutorials. Overall, EasyFlow serves as a simple-to-use tool for inexperienced users with little coding experience to use locally, as well as a platform for advanced users to further customize for their own needs.

## Background

Flow cytometry enables quantitative high-throughput fluorescence measurements in small particles and single cells and has been widely used in immunology, microbiology, synthetic biology, and many other fields. Different flow cytometry machines were developed over the years, with distinct features and utilizing different technologies [1]. However, the FCS file standard (defined by the International Society for Advancement of Cytometry (ISAC) [2, 3]) has been universally adopted as a common output format across all devices. This standardization enormously facilitates the data exchange between different machines and labs.

A diverse spectrum of software solutions has been developed to analyze flow cytometry from FCS files (Table 1). When comparing these tools, several key features are important to highlight. First, the analysis of flow cytometry data depends heavily on visual user interactions. These include viewing different histograms or scatter plots and determining visually gating strategies to extract specific subpopulations of cells. In addition, while a core set of operations is used routinely, flexibility for user customization is important as well. Finally, platform independence, availability, maintenance, and software cost are additional aspects that differ between existing solutions. Current tools can be roughly categorized into three categories—proprietary softwares, open-source libraries, and online analysis tools. Proprietary software, e.g., FlowJo (BD Bioscience) and FCS Express (De Novo Software), provides full-feature powerful analyzing tools. However, such software can be costly and lack transparency and flexibility for user customization. Notably, there are several tools with graphical user interfaces (GUIs), including Flowing Software, Cyflogic, and FCSalyzer, that are freely available for academic users. However, few of them are open-source or capable of further modification and transparency. Some of them are not actively maintained, and they are often operating only on limited platforms (e.g., Windows only). Open-source libraries (usually written in R or Python) provide superior flexibility but require users to have previous, and usually significant, experience in coding. A major weakness of these tools is the lack of visual interactions that are required for many common routine analysis tasks. Online analysis tools can be either commercial (e.g., CellEngine by Cell Carta) or freely available (e.g., Floreada.io). These tools are easy to use across platforms and require no installation. However, big datasets present significant challenges to the routine use of these tools, and repeatability on the same dataset is not always guaranteed, as the host can update or change the service without notice.

**Table 1:** Comparison of currently available software for flow cytometry analysis.

Software	Code availability	Prerequisite	Free	GUI	Platform available^1^	Last updated^2^
EasyFlowQ	Available on Github	None^3^	Yes	Yes	All	2024
EasyFlow	Available on Github	Matlab	Yes	Yes	All	2024
FLowJo	Proprietary	None	No	Yes	Windows & MacOS	2024
FCS Express	Proprietary	None	No	Yes	Windows & MacOS	2024
Flowing Software	Not available	None	Yes	Yes	Windows	2013
Cyflogic	Not available	None	Hybrid^4^	Yes	Windows	2008
FCSalyzer	Open source; not on Github	Java	Yes	Yes	All	2021
FlowCal^5^	Available on Github	Python	Yes	No	All	2021
FlowCytometryTools	Available on Github	Python	Yes	Limited^6^	All	2022
fcm	Available on Github	Python	Yes	No	All	2015
floreada.io	Proprietary	None	Yes	Yes	As website	2024
CellEngine	Proprietary	None	No	Yes	As website	2024

Notes: 1) “All” stands for “available on Windows, MacOS and Linux”; 2) As of June 2024; 3) Require Python on Linux; 4) Cyflogic provides a free license for academic users, with slightly limited functionality; 5) EasyFlowQ’s core processing functions are built on FlowCal [12]; 6) GUI is only available for generating scripts for gating.

Here we present the Matlab-based EasyFlow (github.com/AntebiLab/easyflow) and its derivative standalone Python EasyFlowQ (ym3141.github.io/EasyFlowQ/), which are open source user-friendly GUI, can be run on multiple platforms (Windows, MacOS and Linux), and require no coding knowledge. Both versions were designed to enable a simple tool for viewing, gating, and analyzing flow data while allowing further higher-level analysis using the full power of the corresponding scripting language. Being open source, these tools provide transparency in data processing and allow further customization and collaborative improvements for advanced users. Due to its functionality, EasyFlow was widely used in publications [4–11]

## Results

**Fig 1:**
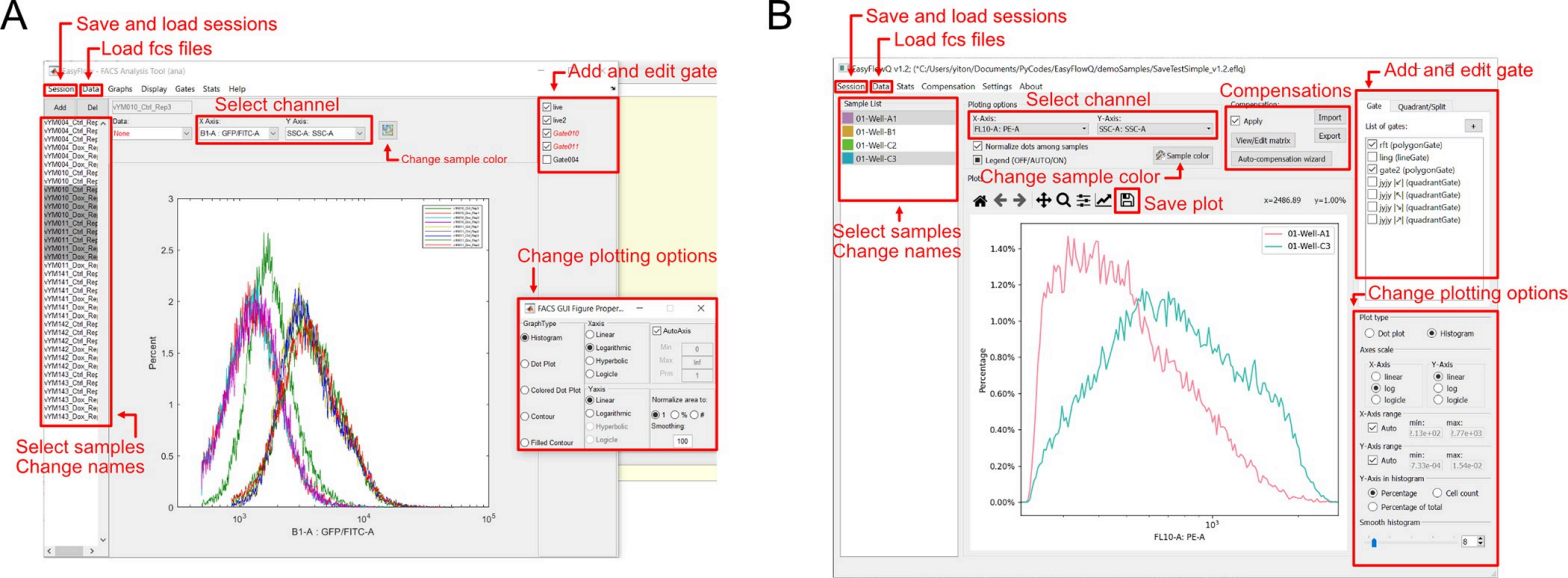
Screenshots of the EasyFlow (Matlab) (A) and EasyFlowQ (Python) (B), with basic functions including managing FCS files, plotting and gating, annotated.

EasyFlow is an open-source software designed as an intuitive tool for visual-based analysis of single-cell flow cytometry data. Its main goal is to simplify the identification and extraction of the proper subset of events for further analysis, as well as to generate the standard histograms and scatter plots. Importantly, EasyFlow provides the capacity to extract the resulting data and key statistical parameters for downstream secondary analysis. To serve this goal, EasyFlow utilizes a simple, plot-centric user interface (Fig 1A and 1B) featuring a single plotting region at the center of the GUI, with most used functions, including sample management, channel selection, gating, plotting options, etc. exposed on the top layer. EasyFlow supports the basic functions for analyzing FCS files: loading and parsing FCS files; plotting histogram, scatter, density, and contour (Matlab version only currently); visually creating and editing 2D and 1D gates; exporting raw data, auto and manual compensation, statistical analysis, etc. We also programmed convenient features such as batch sample renaming and session saving. Statistical features for each gated population can be computed and exported for further analysis. EasyFlow is implemented in both Matlab and Python programming environments, providing three main characteristics: capacity for exporting and interacting with raw data, cross-platform and standalone capability, and flexibility for further community development.

### Exporting and interacting with raw data

In addition to exporting statistics, we designed both versions of EasyFlow to be able to export the processed data (e.g. gated samples) for further in-depth processing. In EasyFlow (Matlab), data can be exported into the Matlab working space for further processing with Matlab scripts or as an Excel file. In EasyFlowQ (Python), the data are exported as comma-separated values (.csv) files that can be conveniently accessed by commonly available programs (e.g. Excel) or programming scripts (e.g., Python and R). These abilities allow users to further analyze their data with great flexibility.

### Cross-platform and standalone capability

EasyFlow was designed for cross-platform usage. The original EasyFlow (Matlab) runs on standard Matlab installation (versions later than 2017a). EasyFlowQ (Python) runs on standard Anaconda installation (based on Python 3, version later than 3.7 recommended). Both softwares are available on all three major operating systems (Windows, MacOS, and Linux). Additionally, we packaged EasyFlowQ using Pyinstaller and InstallForge (Windows) or create-dmg (MacOS) into installers to further eliminate the requirement of installing additional software (packages). These installers can be used on either Windows or MacOS to create standalone software with a similar experience as most mainstream applications.

### Flexibility for further community development

We built both versions of EasyFlow to allow further development from community contribution. More specifically, we ensured that the source code could be run by the standard installation of Matlab (EasyFlow) or the popular Python installation Anaconda in its default state (EasyFlowQ). For the latter, we further provided a list (yaml file) of recommended packages that can be imported within an existing Anaconda installation. We also used Matplotlib and PyQt5, two of the most popular packages in the Python programming community, for plotting and UI framework, respectively. Furthermore, both versions of EasyFlow are hosted on Github, making it possible to benefit from the well-established, community-driven, open-source software development model.

### Example analysis workflow of a mixed population of immune cell lines

**Fig 2:**
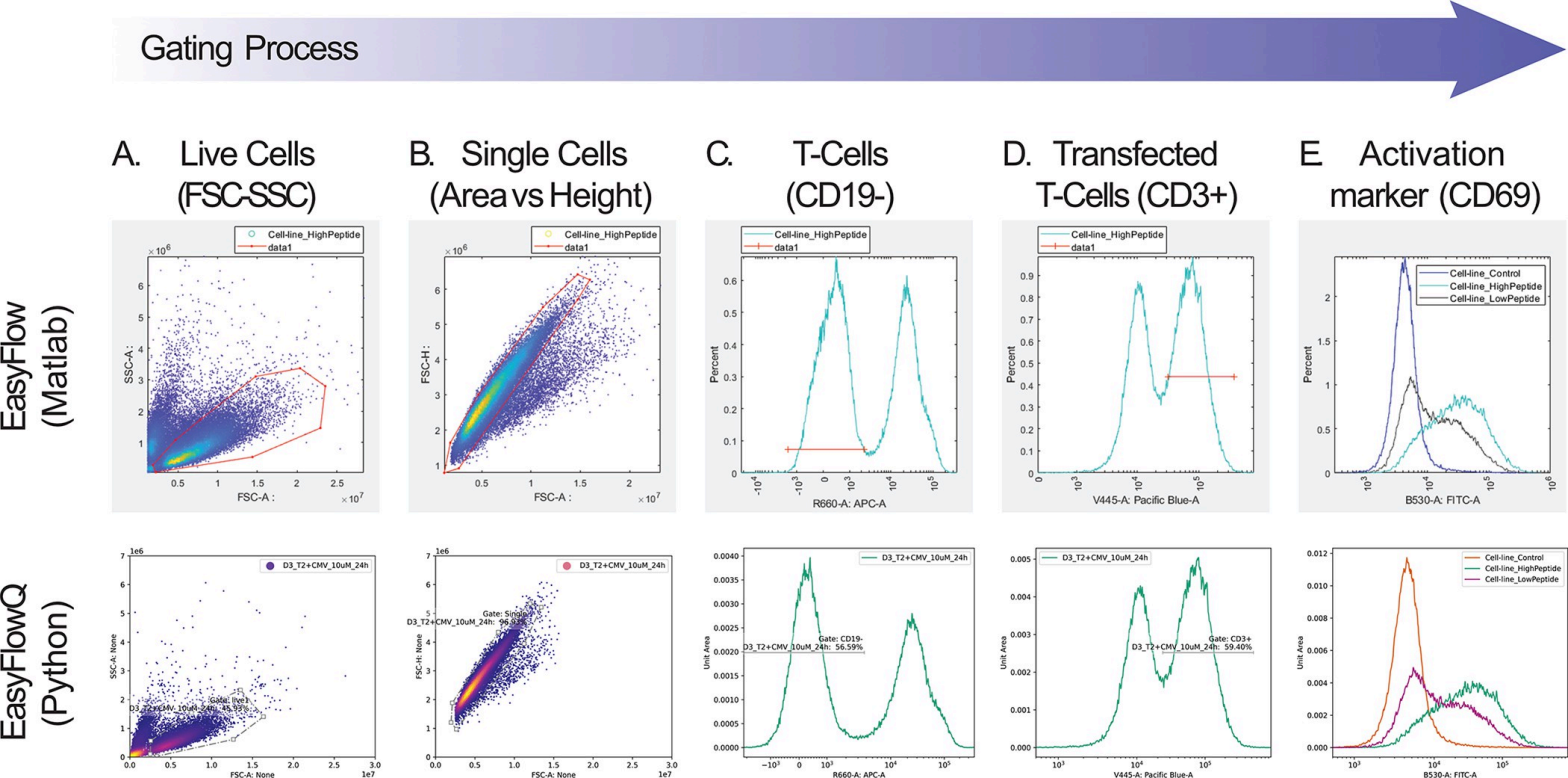
Workflow of T cell activation analysis. T cells (Modified Jurkats, see Methods) were co-cultured with antigen-presenting cells (T2 line, B cells) and their cognate peptide to induce T cell activation. Cells were stained with antibodies to CD19 (B cell marker), CD3 (T cell receptor subunit), and CD69 (T cell activation marker). To gate live cells (A) and single cells (B), cells are plotted using the “Colored Dot Plot’’ option to visualize cell density and identify the sub-populations in the data. Cells are gated using a polygonal 2-dimensional gate, allowing to set the required gate to select for the desired population of cells. Next, a histogram display is used to identify the T cells and remove the B cells (C) and to identify T cells expressing CD3 that can respond to the added peptide (D). Using a 1-dimensional gate, we select the desired cells by choosing the range of values for the corresponding marker within a bi-modal population. In EasyFlow, gates are defined globally so that even if created for a single sample, gates can be applied to all samples in the analysis. In this way, the sequence of gates is applied to all samples in the analysis, enabling the comparison between different conditions. Finally, the percentage of activated cells as determined by the expression of CD69 is examined on the gated live single peptide-sensitive T cells. The percentage of CD69-expressing cells under three conditions: low, high, and no added peptide is examined (E). In all panels, the top row shows the EasyFlow (Matlab) UI, while the bottom row shows the EasyFlowQ (Python) UI.

Here, we demonstrate a workflow for analyzing T cell activation in a co-culture of T cells (modified Jurkat) and B cells (T2) presenting the cognate peptide (Fig 2). We initially analyzed a density scatter plot of the FSC-A vs SSC-A parameters (related to cell size and cell granularity, respectively) using the software. Applying a gating tool allows us to define a polygon for selecting the live cells sub-population. We similarly removed cell aggregates using the relationship between the FSC-A vs FSC-H parameters (total signal vs peak signal). By changing to a histogram view, we filtered out the B cells by selecting CD19- cells (APC-A channel) and further focused on the fully modified Jurkat cells expressing CD3 (Pacific Blue-A channel). When creating a gate using the EasyFlow software, it is defined globally and thus can immediately be applied to any sample. Here, we set up the gates using a positive control sample, and further applied them to all samples in the data, showing the activation response across three levels of peptide (Fig 2E). More generally, gates can also be defined by plotting data from multiple samples, allowing the definition of more complex gates based on all needed information in a single window. Furthermore, while gating has been traditionally thought of as purely hierarchical, there is nothing inherently hierarchical in its definition. For example, the order by which gates are applied is completely commutative, as the resulting gated cells are exactly the same regardless of the application order of the gates. Therefore, in EasyFlow, gates are independent objects. In this way, every gate can be applied to every sample and in any order. This allows, for example, to define a specific gate on a marked sub-population, and later then to apply the same gate on the entire population without first applying the gate for the sub-population. We also provide hierarchical statistics (e.g., percentage in each step of gating) in the “Stats” window, which are critical in various analyses.

## Discussion

EasyFlow provides an accessible and simple solution for the analysis of flow cytometry data. It enables standard analysis tools and provides the capacity for in-depth analysis using commonly used scripting languages such as Matlab or Python. Initial flow cytometry data analysis follows a standard pipeline, including standard gating and plotting. However, as high throughput data becomes commonly used across biological fields, more complex experiments arise, requiring additional steps of a project-specific analysis that is tailored to the specific data. As Easyflow is based on commonly used scripting languages, it allows for a direct and seamless integration for more advanced analysis when required. For example, gated data can be exported directly to the programming environment or to a standard data file (CSV or Excel) that can be further analyzed.

Open-source analysis software that is designed to be user-friendly had lots of successful examples in the past in the field of image analysis (ImageJ [13]) and next-generation sequencing (IGV [14]). One key advantage of being open source, especially when hosted on popular version control platforms like Github, is providing transparency of data handling and opening up the possibility of further customization by other researchers. It is our goal that new features suggested by the community will be added to the software, expanding its capabilities in the future.

For everyday users looking for an easy-to-use local FCS analyzer, we recommend EasyFlowQ (python-based), as its standalone packages require no pre-installation of either Matlab or Python, as well as have a user experience similar to other native Windows or MacOS applications. EasyFlow is optimized for users who are familiar with Matlab and need access to the raw data for subsequent analysis with Matlab scripts. Contributions through Github, including forking, reporting issues, and requesting features, are welcome.

## Methods

### T cell activation

T cell activation was measured on a Jurkat cell line transduced with a class I A2 restricted TCR specific for CMV peptide and lacking endogenous TCR. Cells were activated by co-culture with the T2 cell line (B cells) as antigen-presenting cells, cultured at a 1:1 ratio, together with the cognate CMV peptide. To examine the level of activation, cells were cultured for 24h together with a high level of peptide (10uM), a low level of peptide (10 nM), or no added peptide. Following 24h of culture, cells were stained with antibodies against CD19 (B cells recognition), CD3 (TCR expressing T cells), and CD69 (T cell activation marker) and analyzed using flow cytometry.

### Flow analysis

Cells were measured using the NovoCyte Quanteon flow cytometer (Agilent). The resulting data was analyzed in parallel by both EasyFlow (antebilab.github.io/easyflow/) on Matlab R2023a and EasyFlowQ’s version 1.5 (github.com/ym3141/EasyFlowQ/releases/tag/v1.5.6). A step-by-step walkthrough of the EasyFlowQ’s analysis is available at ym3141.github.io/EasyFlowQ/Tutorial/.
